# Synaptic Transmission and Plasticity in an Active Cortical Network

**DOI:** 10.1371/journal.pone.0000670

**Published:** 2007-08-01

**Authors:** Ramon Reig, Maria V. Sanchez-Vives

**Affiliations:** Instituto de Neurociencias de Alicante, Universidad Miguel Hernandez-CSIC, San Juan de Alicante, Spain; Duke University, United States of America

## Abstract

**Background:**

The cerebral cortex is permanently active during both awake and sleep states. This ongoing cortical activity has an impact on synaptic transmission and short-term plasticity. An activity pattern generated by the cortical network is a slow rhythmic activity that alternates up (active) and down (silent) states, a pattern occurring during slow wave sleep, anesthesia and even *in vitro*. Here we have studied 1) how network activity affects short term synaptic plasticity and, 2) how synaptic transmission varies in up *versus* down states.

**Methodology/Principal Findings:**

Intracellular recordings obtained from cortex *in vitro* and *in vivo* were used to record synaptic potentials, while presynaptic activation was achieved either with electrical or natural stimulation. Repetitive activation of layer 4 to layer 2/3 synaptic connections from ferret visual cortex slices displayed synaptic augmentation that was larger and longer lasting in active than in silent slices. Paired-pulse facilitation was also significantly larger in an active network and it persisted for longer intervals (up to 200 ms) than in silent slices. Intracortical synaptic potentials occurring during up states *in vitro* increased their amplitude while paired-pulse facilitation disappeared. Both intracortical and thalamocortical synaptic potentials were also significantly larger in up than in down states in the cat visual cortex *in vivo*. These enhanced synaptic potentials did not further facilitate when pairs of stimuli were given, thus paired-pulse facilitation during up states *in vivo* was virtually absent. Visually induced synaptic responses displayed larger amplitudes when occurring during up *versus* down states. This was further tested in rat barrel cortex, where a sensory activated synaptic potential was also larger in up states.

**Conclusions/Significance:**

These results imply that synaptic transmission in an active cortical network is more secure and efficient due to larger amplitude of synaptic potentials and lesser short term plasticity.

## Introduction

The cortical network *in situ* is permanently active, its patterns of activity varying depending on the waking or sleep states [1]–[3]. During sleep, the activity is mostly oscillatory and generated by the recurrent connections existing between cortical neurons [4] and within the thalamocortical loop [5]–[7]. The activity in the cortical network -originated either from sensory inputs or emergent from the recurrent connectivity- has an impact on different properties of the network itself, such as on the intrinsic properties of neurons [8], [9] and on the short term plasticity of its synaptic connections [10]–[15].

The functional state of the cortex also affects synaptic transmission and sensory processing. During slow, rhythmic activity in the cortical network the activity is organized in up or activated states, depolarized and rich in synaptic noise and down, hyperpolarized and synaptically silent states [16]–[21]. Different studies have analyzed how the state of activation of the cerebral cortex affects synaptic responsiveness [22] and sensory transmission [13], [23]–[25], yielding diverse results that may reflect differences between species, cortical areas, experimental design or interpretation.

In the study that we present here two main aspects related to synaptic transmission and activity on the cortical network have been considered: 1) short term synaptic plasticity under different levels of activity in the network and 2) synaptic transmission and plasticity during up *versus* down states. The study has been carried out in different preparations and different stimuli have been used: electrical activation of intracortical connections in visual cortex *in vitro* and *in vivo* and thalamocortical connections *in vivo*, visually evoked synaptic potentials in visual cortex, and whisker evoked responses in barrel cortex.

## Methods

### Slices preparation

The methods for preparing cortical slices were similar to those described previously [19]. Briefly, cortical slices were prepared from 2- to 6-month-old ferrets of either sex that were deeply anesthetized with sodium pentobarbital (40 mg/kg) and decapitated. Four hundred-micrometer-thick coronal slices of the visual cortex were cut on a vibratome. A modification of the technique developed in [26] was used to increase tissue viability. After preparation, slices were placed in an interface-style recording chamber (Fine Sciences Tools, Foster City, CA) and bathed in what we refer to in the Results as “classical” ACSF containing (in mM): NaCl, 124; KCl, 2.5; MgSO_4_, 2; NaHPO_4_, 1.25; CaCl_2_, 2; NaHCO_3_, 26; and dextrose, 10, and was aerated with 95% O_2_, 5% CO_2_ to a final pH of 7.4. Bath temperature was maintained at 34–35°C. Intracellular recordings were initiated after 2 hr of recovery. In order to induce spontaneous rhythmic activity, the solution was switched to “in vivo-like” ACSF containing (in mM): NaCl, 124; KCl, 3.5; MgSO_4_, 1; NaHPO_4_, 1.25; CaCl_2_, 1-1.2; NaHCO_3_, 26; and dextrose, 10.

### Animal preparation for *in vivo* recording. Cat visual cortex

Intracellular recordings *in vivo* from the primary visual cortex of cats were obtained following the methodology that we have previously described [27]. In short, adult cats were anesthetized with ketamine (12–15 mg/kg, i.m.) and xylazine (1 mg/kg, i.m.) and then mounted in a stereotaxic frame. A craniotomy (3–4 mm wide) was made overlying the representation of the area centralis of area 17. To minimize pulsation arising from the heartbeat and respiration a cisternal drainage and a bilateral pneumothorax were performed, and the animal was suspended by the rib cage to the stereotaxic frame. During recording, anesthesia was maintained with i.m. injections of both ketamine (7 mg/kg) and xylacine (0.5 mg/kg) every 20–30 min. If visual responses were studied, the animal was paralyzed with norcuron (induction 0.3 mg/Kg; maintenance 60 µg/kg/h) and artificially ventilated. The heart rate, expiratory CO_2_ concentration, rectal temperature, and blood O_2_ concentration were monitored throughout the experiment and maintained at 140–180 bpm, 3–4%, 37–38°C, and >95%, respectively. The EEG and the absence of reaction to noxious stimuli were regularly checked to ensure an adequate depth of anesthesia. After the recording session, the animal was given a lethal injection of sodium pentobarbital.

Ferrets, cats and rats were cared for and treated in accordance with the Spanish regulatory laws (BOE 256; 25-10-1990) which comply with the EU guidelines on protection of vertebrates used for experimentation (Strasbourg 3/18/1986).

### Rat barrel cortex

Four adult Wistar rats (250–300 grs) were used for recordings in S1 cortex. Anesthesia was induced by intraperiotoneal injection of ketamine (100 mg/kg) and xylacine (8–10 mg/kg). The animals were not paralyzed. The maintenance dose of ketamine was 75 mg/kg/h. Anesthesia levels were monitored by the recording of low-frequency electroencephalogram (EEG) and the absence of reflexes. Rectal temperature was maintained at 37°C. Once in the stereotaxic apparatus, a craniotomy (2×2 mm) was made at coordinates AP –1 to -3 mm from bregma, L 4.5–6.5 mm [28]. After opening the dura, extracellular recordings were obtained with a tungsten electrode (FHC, Bowdoinham, ME, USA). For stability and to avoid desiccation agar (4%) was used to cover the area. Extracellular recordings were used to adjust whisker stimulation (see below) and to monitor the occurrence of slow oscillations. Intracellular recordings (see below) were obtained within 1 mm from the extracellular recording electrode. ***Whisker stimulation.*** A puff of air given through a 1 mm tube placed in front of the whiskers (10–15 mm) was used for stimulation. The air puff (10 ms) was controlled by an stimulator and delivered by a Picopump (WPI, Sarasota, FL). The whisker displacement was not monitored and the time 0 for the stimulus was taken as the initiation of the air puff.

### Recordings and stimulation

Sharp intracellular recording electrodes were formed on a Sutter Instruments (Novato, CA) P-97 micropipette puller from medium-walled glass and beveled to final resistances of 50–100 MΩ. Micropipettes were filled with 2 M KAc. Recordings were digitized, acquired and analyzed using a data acquisition interface and software from Cambridge Electronic Design (Cambridge, UK). Electrical stimulation (0.1 ms, 10–300 µA) was delivered by means of a WPI A-360 stimulus isolation unit (Sarasota, FL) that prevents electrode polarization. *In vitro*, a concentric bipolar stimulating electrode (FHC, Bowdoinham, ME, USA) was placed in layer 4 and the postsynaptic neurons were recorded in layer 2/3. *In vivo* thalamocortical (TC) or intracortical (IC) fibers were stimulated with bipolar electrodes made of sharpened tungsten wires. For details on how TC stimulation was delivered see [15]. IC stimulation was delivered at 500–1500 µm from the intracellularly recorded neuron. Both *in vivo* and *in vitro*, and both in TC and IC, the intensity of the stimulation was adjusted to achieve a stable PSP amplitude, which at the population level ranged between 2 and 7 mV. Criteria for monosynaptic connections were: reliably evoked synaptic potentials (no failures) of constant amplitude and shape and with a constant latency (jitter<1 ms) and latency of 1.3–3 ms. To confirm that the PSPs were excitatory, their amplitude was often examined at different membrane potentials. However, since we cannot rule out a possible participation of reversed IPSPs, we refer to the synaptic response as PSPs.

### Visual stimulation

The location of the neuronal receptive field and orientation preference were first explored with a handheld projector. Next, visual stimuli were delivered with a computer monitor (Vision Master Pro 450, 90 Hz refresh rate) and triggered from Spike 2 (Cambridge Electronic Design, Cambridge, UK). A white bar of preferred orientation at ±50% contrast against the background was flashed for 20 ms over the receptive field every 2 sec. The size of the bar was adjusted in order to induce a small (1–7 mV) synaptic response (see below), comparable to the ones evoked by electrical stimulation. Visual triggers were later sorted off line as occurring during up or down states. During stimulation protocols aimed at examining short term plasticity, neurons were hyperpolarized to −80 mV±6 mV to prevent action potential firing.

### Analysis

The amplitude of the PSPs was measured at the peak, which had latencies between 3–6.5 ms. As has been described before regarding these same connections [15] PSP slope and amplitude were highly correlated. Paired pulse plasticity was studied by inducing pairs of PSPs evoked with intervals of 10, 25, 50, 75, 100, 200 and 400 ms. When comparing between up and down states, normalization was always done within each cell with respect to the amplitude of the first PSP of the pair during down states. Then, the normalized values for individual neurons were averaged to provide population data, and these were the values represented in the bar diagrams in the different figures. In the text the average of the non-normalized PSPs on each condition are also given. To quantify paired pulse plasticity, the amplitude of the second PSP was divided by the one of the first PSP within the same condition and for each cell. For the PSPs pairs, often the proportion between second with respect to the first PSP has been represented. Data are given in the text as mean±s.d. Error bars in the figures correspond to the s.e.m.

## Results

Here we include data obtained from active, oscillatory brain slices as well as from recordings during cortical slow oscillations in anesthetized animals. All recordings included in this study were obtained from the visual cortex of the ferret (*in vitro*), cat (*in vivo*) and from barrel cortex of the rat (*in vivo*). Twenty-nine neurons recorded from ferret cortical slices are included in this study (22 regular spiking (RS); 5 chattering (CH) and 2 intrinsic bursting (IB)), 27 neurons from cat visual cortex *in vivo* (14 RS; 5 CH; 2 IB; 3 fast spiking (FS); plus 3 non classified) and 14 neurons from rat barrel cortex *in vivo* (12 RS; 1 CH; 1 IB). Synaptic potentials were evoked by electric shocks (intracortical or thalamocortical connections) or by means of sensory (visual or whisker) stimulation. The main results are: 1) Synaptic potentials show more paired pulsed facilitation and synaptic augmentation in active than in silent cortical networks and 2) Synaptic potentials occurring during up or activated states of the cortex increased their amplitude with respect to those occurring during down states.

### Synaptic potentials and facilitation in active and silent cortical slices

**Figure 1:**
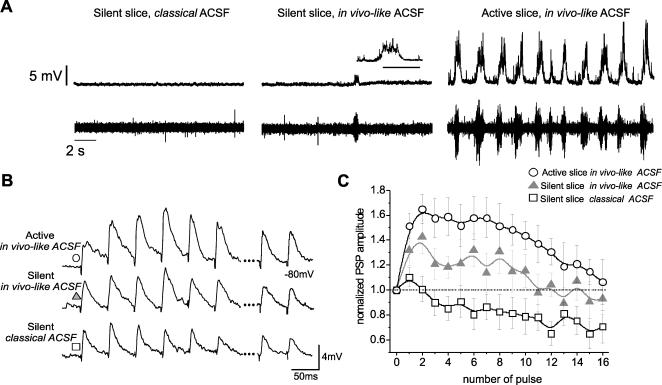
Short term synaptic plasticity of intracortical PSPs in active *versus* silent slices. A. Intra (top) and extracellular (bottom) recordings illustrating cellular and network activity repectively in the three conditions depicted in B and C. The recordings correspond to the same neuron and location in the slice. On the left, the slice is silent and it is immersed in the so-called *classical* ACSF (for composition of ACSF see Methods). In the middle traces the slices are in *in vivo-like* ACSF but spontaneous rhythmic activity has not developed yet, although occasional bursts of activity can be observed. The traces on the right show organized up and down states observable in both the recordings, in the case of the intracellular (top) the neuron being kept subthreshold. B. Raw traces of 16 PSPs recorded from a layer 2/3 neuron in the visual cortex induced by repetitive electrical stimulation (20 Hz) of layer 4. The first 6 and the last 2 PSPs of the 16 are shown. The same neuron was recorded while in three different funcional states of the cortical network: 1) top trace, PSPs recorded from an active, oscillatory slice, 2) middle trace, PSPs recorded from silent slices in ‘in vivo-like’ ACSF, and 3) bottom trace, PSPs recorded from silent slices in ‘classical’ ACSF. C. Averaged and normalized PSPs amplitudes for the same three experimental conditions described in A: active, oscillatory slice in ‘in vivo-like’ ACSF (○), silent slice in ‘in vivo-like’ ACSF (▴) and silent slice in ‘classical’ ACSF (□). Each point corresponds to the average of several cells (between n = 5 and 17) and the error bar represents±s.e.m. The plotted values correspond to the normalized ones with respect to the amplitude of the first PSP (at 0 sec). The connecting curve between points is a B-spline. All the recordings included in this graph were from slices that have been kept at least 20 min in the aforementioned solutions.

Intracortical synaptic potentials were evoked in layer 2/3 neurons by electrical stimulation of layer 4 in ferret visual cortex slices, as in [15]. Repetitive stimulation of these synaptic connections showed different types of short term synaptic plasticity depending on the ongoing activity in the network. Synaptic plasticity was compared in two different functional situations of the cortical network: in silent slices (without spontaneous activity) *versus* in active (oscillatory) slices (Fig. 1A; see Methods) .

Silent cortical slices were those in which no spontaneous, rhythmic activity occurred. In these slices neither intra nor extracellular multiunit recordings would detect spontaneous network activity (Fig. 1A, left and middle). Activity, thus, should be evoked by external means: intracellular injection of current, electrical or chemical stimulation. Silent slices can be recorded in *classical* or *in vivo-like* ACSF(see Methods), in the later case before activity has developed, or in slices in which organized activity does not occur, as described in [15]. After a period of time (30–45 min) in *in vivo-like* ACSF rhythmic oscillatory would appear (Fig. 1A, right hand side), highly similar to the one occurring during slow wave sleep [19].

Repetitive activation of intracortical connections induced a significantly larger synaptic augmentation in active than in silent slices during series of 16 pulses at 20 Hz (Fig. 1C). If the stimulation persisted for more than 15 pulses, augmentation gave way to synaptic depression, which was lesser in active than in silent slices [15]. Synaptic potentials in silent slices in *classical* ACSF hardly showed any enhancement with repetitive stimulation but only synaptic depression from the very first pulses, probably due to the higher calcium concentration of this solution. However, synaptic potentials recorded from silent slices in *in vivo-like* ACSF showed some augmentation during the first 10 pulses, although lesser than the one in active slices maintained in ACSF with the same ionic composition (Fig. 1B, C). Therefore, the difference in synaptic plasticity observed between silent and active slices, both in the same ACSF (*in vivo-like*) can only be attributed to the difference in ongoing activity in the network. From this first section of the results we conclude then that the studied intracortical connections have a stronger tendency to display augmentation in an active than in a silent cortical network, and as we showed in a previous study, less tendency to depress [15].

**Figure 2:**
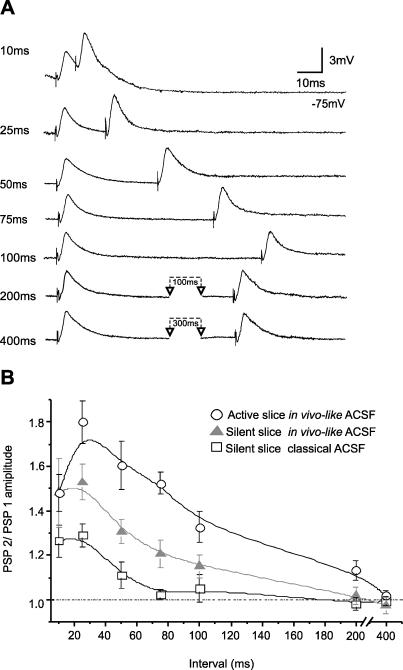
Paired pulse facilitation in active *versus* silent slices. A. Raw traces of a paired pulse protocol in a layer 2/3 neuron. Pairs of PSPs were evoked with different intervals (10–400 ms) by electrical stimulation of layer 4 in a silent slice in ‘in vivo-like’ ACSF. B. Relative amplitude of the second PSP with respect to the first one (PSP2/PSP1) represented for different intervals (10–400 ms) in three different functional states of the slices: active, oscillatory slice in ‘in vivo-like’ ACSF (n = 10; ○), silent slice in ‘in vivo-like’ ACSF (n = 11; ▴) and silent slice in ‘classical’ ACSF (n = 12; □). The error bar represents±s.e.m.

Next we explored paired-pulse plasticity for different intervals (10 to 400 ms) in between stimuli (Fig. 2A). In general, synaptic potentials separated by shorter intervals showed larger facilitation, and this facilitation decayed for longer intervals until both synaptic potentials in the pair were of the same amplitude (Fig. 2). Paired-pulse facilitation was compared for the same functional states of the cortex as the ones presented above (active and silent slices, the latter both in *classical* and *in vivo-like* ACSF). In all cases maximum facilitation was achieved when the interval between the first and the second synaptic potentials was of 25 ms (PSPs). However, in much of the data and many of the figures reported in these results we will provide and use for comparisons the relative amplitudes for pairs of stimuli separated by 50 ms. Facilitation is still quite prominent with a 50 ms interval (Fig. 2B), and this larger interval assures that the second PSP does not overlap the first one, therefore facilitating the measurement.

PSPs in active slices showed larger paired pulse facilitation, with an average increase of the second PSP with respect to the first one of 1.8 times (n = 7) for a 25 ms interval and 1.6 times (n = 9) for 50 ms interval. PSPs separated by up to 200 ms still showed significant facilitation in active slices (Fig. 2B). In silent slices–no spontaneous activity-there was less synaptic facilitation than in active slices for every time interval (Fig. 2B; n = 19 neurons). Neurons in silent slices maintained in *classical* ACSF (n = 7) displayed the lowest values of synaptic facilitation and no facilitation was observed for intervals longer than 75 ms. Neurons in silent slices in *in vivo-like* ACSF (n = 12) displayed larger synaptic facilitation than silent slices in *classical* ACSF, facilitation that was maintained for longer intervals between pulses (Fig.2B). However, this facilitation was still significantly lower than the one occurring in active, oscillatory slices. Therefore, the difference observed in facilitation between silent slices in *classical* versus *in vivo-like* ACSF should be attributed to the difference in ionic composition of the ACSF. But differences in facilitation detected between silent and active slices, both in the same (*in vivo-like*) ionic environment, can only be attributed to the activity itself. Therefore, layer 4 to layer 2/3 intracortical connections in the visual cortex showed a larger paired pulse facilitation and of longer duration when the network had rhythmic spontaneous activity than when it was silent.

**Figure 3:**
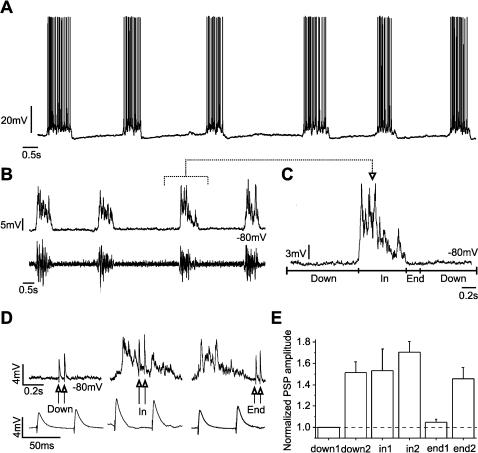
Paired pulse facilitation in up *versus* down states in the cortex *in vitro.* A. Six up states interspersed with down, silent states recorded from a layer 2/3 neuron in primary visual cortex of the ferret *in vitro*. B. Four up states (top trace) and the corresponding multiunit activity recorded in the vicinity of the neuron (bottom trace). The intracellular recordings were kept subthreshold by means of current injection. C. Expanded up state illustrating the time segments that will be used to sort out the time of occurrence of the PSPs: *Down* (down state periods excluding the 200 ms following an up state), *In* (up states) and *End* (200 ms following the occurrence of the up state). D. Raw traces of a pair of PSPs occurring during the *Down*, *In* and *End* periods (top trace). Averaged paired PSPs for the same neuron during the 3 periods with respect to the occurrence of the up states: *Down, In* and *End* (bottom trace) (n = 8 PSPs have been averaged for each segment). E. Bar diagram illustrating the amplitude of the first and second PSP of the pair during the 3 time segments (*Down, In* and *End*) for intracortical synapses recorded *in vitro*. All the PSPs have been normalized with respect to the first PSP of the pair in the down states (n = 9 neurons).

Paired synaptic potentials recorded in active, oscillatory slices, and included above in this section, were recorded during the intervals in between oscillations or down states (see Fig 3B). However, once the network is active, how does the reverberatory network activity that occurs during up states affect synaptic transmission and plasticity?

### Cortical synaptic transmission and plasticity during up and down cortical states in vitro *and* in vivo

To answer this question activation of layer 4 to layer 2/3 synaptic connections was induced at different times relative to the occurrence of up states, up states refering to those periods during which the cortical network remains depolarized and ‘activated’ due to reverberatory activity in cortical circuits (Fig. 3A, B). Pairs of PSPs were evoked during down states (*Down*), during up states (*In*) and in the next 200 ms following an up state (*End*) in n = 9 neurons (Fig 3C,D) recorded from ferret visual cortex *in vitro*. The average value of the first PSP was 2.6±1.2 mV (n = 9) when occurring during down states. In contrast, the average amplitude of the first PSP during up states had an increased amplitude to an average value of 4.0±1.2 mV (n = 9), significantly larger (t-test; p<0.03). This increase in the amplitude of the first PSP was not corrected for the membrane potential value, implying that due to the reversal potential of glutamate receptors (around 0 mV) the amplitude of a PSP evoked at a more depolarized potential should be smaller. The average amplitude of the up states *in vitro* in our preparation was around 10 mV. Therefore, if we estimate this difference for a pure AMPA excitatory potential, a depolarization of 10 mV would decrease the amplitude of the EPSP in ×0.17 (based on AMPA I-V taken from [29]. As a result, our observation of an average increase of 1.53 times in the PSP amplitude during up states is slightly underestimated due to the difference in membrane potential. As we will demonstrate below, it has been a consistent finding in our studies that synaptic potentials occurring during up states were of significant larger amplitude than those occurring during downstates, not only for electrically evoked intracortical and thalamocortical synaptic potentials, but also for the visually and whisker stimulation evoked ones.

During down states in the cortical slices almost all (8 out of 9) of the recorded neurons showed significant paired pulse facilitation (which was on average ×1.5 for intervals of 50 ms; see Figure 3E). However, paired pulse facilitation during up states was not significant, probably due to the fact that the first PSP was already enhanced. Pairs of PSPs evoked at the end of an up state showed again paired pulse facilitation, similar to the one observed during down states (Fig. 3E).

**Figure 4:**
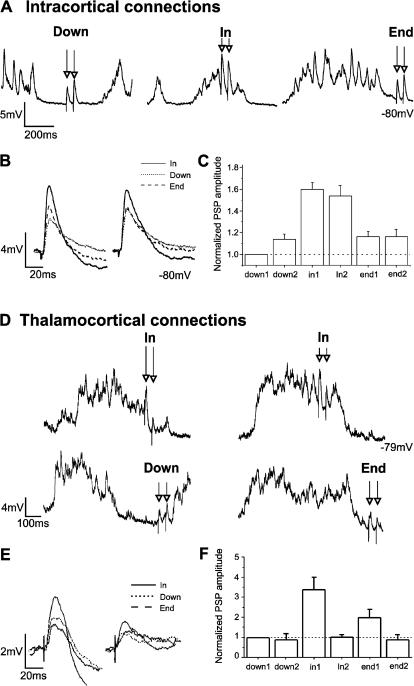
Paired pulse facilitation in up *versus* down states in the cortex *in vivo.* A. Raw traces of pairs of intracortical PSPs occurring during *Down* (down states), *In* (up states) and *End* (200 ms following up states) periods during an intracellular recording *in vivo* in the cat primary visual cortex. B. Averaged amplitudes of the first and second intracortical PSPs in the pair occurring in the three situations (*Down, In* and *End*) shown in A. C. Bar diagram illustrating the relative amplitudes of first and second intracortical PSPs in the three periods (*Down, In* and *End*) all of them normalized with respect to the amplitude of the first PSP in the down state, which is therefore represented as amplitude 1 (n = 9 neurons). D. Two different examples of thalamocortical PSPs occurring during the up states (*In* period; top traces): one triggering the initiation of a down state (left), and both PSPs occurring during the up state (right). The bottom traces display the PSPs occurring during the down state (*Down* period; left) and right after the end of an up state (*End* period; right). All the traces in D correspond to a recording from the same neuron. E. Averaged amplitudes of the first and second thalamocortical PSPs in the pair occurring in the three situations (*Down, In* and *End*) shown in D. F. Bar diagram illustrating the relative amplitudes of first and second thalamocortical PSPs in the three conditions (*Down, In* and *End*) all of them normalized with respect to the amplitude of the first PSP in the down state (n = 9 neurons).

The increased amplitude of the first intracortical PSP in up *versus* down states *in vivo* (Fig. 4A, B,C) was very similar to the one described *in vitro*, with an average of ×1.6 times larger amplitude of the PSPs evoked during up states. PSPs occurring in the next 200 ms following an up state were as well significantly larger (×1.18) than the ones occurring during down states. Regarding the absolute –non-normalized- values, the average value of the PSPs during down states *in vivo* was 4.3±1.2 mV (n = 9) and 5.0±1.2 mV (n = 9) (first and second PSPs in the pair respectively). Facilitation of paired pulses (50 ms interval) during down states *in vivo* was (Fig. 4C) less prominent than the one *in vitro* (Fig. 3E). During up states, paired intracortical synaptic potentials *in vivo* displayed neither significant facilitation nor depression for 50 ms intervals (Fig. 4C).

### Thalamocortical synaptic transmission and plasticity during up and down cortical states in vivo

Monosynaptic thalamocortical potentials were evoked by electrical stimulation in the LGN in regions with overlapping receptive fields with the recorded neurons in visual cortex (see Methods; average latency 2.2±0.5 ms) [15]. The average amplitude of the first PSP of the pairs during down states was 1.9±1.1 mV (n = 9). During down states, pairs of synaptic potentials (interval 50 ms) did not show neither significant facilitation nor depression. This result (Fig. 4F) is similar to the one observed for intracortical connections *in vivo* (see above; Fig. 4C) but quite different from the paired pulse facilitation observed for intracortical connections *in vitro* (Fig.3E).

The amplitude of the first PSP in the pair of thalamocortical potentials had an increased amplitude during up states to an average of normalized values of ×3.4 times the amplitude during down states (4.5±2.1 mV; Fig. 4D, E, F). During up states however no facilitation was observed between the first and the second synaptic potentials. On the contrary the average amplitude of the second PSP was decreased with respect to the first one in the pair (1.8±1.1 mV; Fig. 4F), therefore losing all synaptic enhancement and returning to the average amplitude during down states. However this was not always the case, and Fig. 4D illustrates two different cases often observed (from left to right): 1) The electric shocks in the thalamus evoked the end of the up state. This was often the case with the thalamic electric activation (90% of times) but not so with the intracortical activation. In these cases the second PSP was generally smaller than the first one. 2) Both PSPs occurred during the up state (the end of the up state was not induced by the electrical stimulation) and still the second PSP was of smaller amplitude than the first one.

The amplitude of PSPs evoked during the 200 ms following the occurrence of the up state was still increased to an average of the normalized values of ×1.9 times (2.3±0.7 mV) the amplitude of the PSPs occurring during downstates (Fig. 4D,E,F). The second PSP of the pair during this period was also back its original amplitude during down states, therefore losing the effect of the enhancement.

We have also studied the behavior of polysynaptic responses (latencies >3 ms) with respect to the occurrence of up and down states, and we find it not to be different from that of monosynaptic connections (Fig. S1; Supplemental Data).

### Transmission of visual information during up and down cortical states

**Figure 5:**
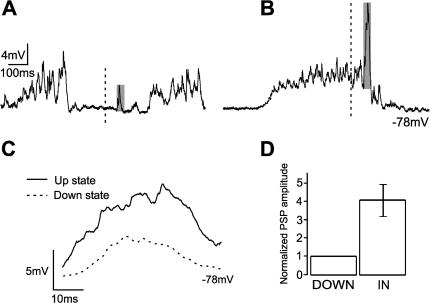
Visually evoked synaptic potentials in up *versus* down states in the cortex *in vivo.* A. Raw trace of a synaptic response (highlighted with a grey box) to a visual stimulus (discontinous line) during a down state. There are up states before and after the visual response. B. Raw trace of the synaptic response activated by the same visual stimulus but now during an up state. C. Average of the visual synaptic responses induced by the same stimulus during up (solid line) and down states (discontinous line). The averages are for 8 visual responses each, and they correspond to the recordings from a unique neuron. D. Relative amplitudes of the visually induced synaptic responses normalized for each neuron with respect to the ones during down states (n = 9 neurons).

Given the prominent increase in amplitude that electrically evoked PSPs showed during up states (see above), we explored how visually evoked synaptic potentials varied by occurring during down *versus* up states. With this purpose, visual synaptic responses were induced by a flashing a bar of optimal orientation within the receptive field of an intracellularly recorded neuron *in vivo* (n = 9). The size of the stimulus was adjusted such that it would evoke a synaptic response of similar amplitude to the ones evoked by electrical stimulation (average of 1.2 mV during downstate; Fig. 5A). The neuronal membrane potential was maintained subthreshold by injecting hyperpolarizing current in order to measure changes in synaptic potential amplitude without evoking action potentials. A problem that we sometimes encountered was that visual synaptic activation induced by visual stimulation could induce an up state by itself [30]. Between 15 and 30 visual stimuli were recorded from each neuron. Visually evoked synaptic responses (or rather, the visual stimulus triggers) were selected into two groups, those occurring during up (Fig. 5B) and down states (Fig. 5A), the ones given during a down state that evoked an up state being excluded. A spike triggered average was done around the time when the visual stimuli occurred for visual responses occurring during both down and up states (Fig. 5C) and the amplitude measured. Averaged visually evoked potentials during down states (1.2±1.0 mV; n = 9) showed an increase in the average PSP evoked during up states to (3.7±3.1 mV; n = 9; p<0.04; Fig. 5D). The value of this increase in the PSP's amplitude was similar to the one observed for the electrically evoked thalamocortical synaptic potentials, and larger than the synpatic enhancement observed for intracortical synaptic potentials.

### Transmission of whisker information during up and down cortical states in rat barrel cortex

A different preparation was used in order to test the transmission of synaptic potentials evoked by natural stimuli during up and down states in a different sensory modality (see Methods). By using this preparation we were able to test if the observations realized in the visual cortex regarding synaptic enhancement during up states could apply to other cortical areas as well. We selected the barrel cortex of the rat to analyze the response to the whisker stimulation during up versus down states. This same preparation has been used by other authors e.g. [23], [25] therefore allowing a direct comparison of the results.

**Figure 6:**
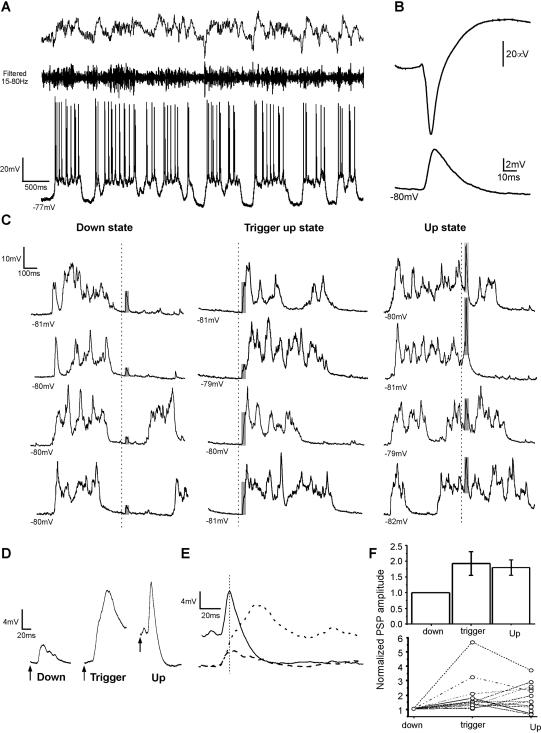
Whisker evoked synaptic potentials in up *versus* down states in the cortex *in vivo.* A. Top trace. Extracellular, unfiltered, recording in the vicinity of the intracellularly recorded neuron in the barrel cortex of the rat. Middle trace. Filtered trace (15–80 Hz) to better illustrate the ocurrence of up states. Bottom trace, intracellular recording of a neuron during slow rhythmic activity. B. Whisker stimulation evoked response. Top, extracellular recording. Bottom, simultaneous intracellular recording. Each response is the average of 20 stimuli. C. Raw traces showing the PSP evoked by a puff of air in three different periods with respect to the occurrence to the up state: during down states (traces to the left), when PSP triggered an up state (middle traces) and when the PSP is evoked during an up state (traces to the right). D. In the same cell, the result of averaging 20 PSPs in each of the three cases shown in B. E. The same averages as in D but overlapped. The discontinous line illustrates where the amplitude measurements were taken, which is at the peak of the PSPs occurring during up and down states. F. Top, bar diagram of the normalized PSP values averaged for 13 neurons. Bottom, normalized values for each of the 12 neurons (an outlier was not represented).

The barrel cortex of a ketamine anesthetized rat (see Methods) showed a consistent alternation between up and down states (Fig. 6A, Fig. S2C,E) as shown by others. A total of 13 intracellular recordings of durations between 6 and 49 min were obtained (n = 4 rats). Alternating up and down states were recorded in all cases (Fig. 6A) in the ketamine anesthetized rats (dose of maintenance 75 mg/kg/h ). A puff of air (10 ms) delivered to the whiskers was adjusted such that it would evoke a synaptic potential of an amplitude 50–100 µA in the field potential, and 5–10 mV in the intracellular recordings with rise time of ≤10 ms (Fig. 6B).

We sorted the synaptic potentials during down states in two types: the isolated ones (Fig. 6C left) and the ones that triggered a new up state (Fig. 6C middle; for additional examples see Fig. S2). The average amplitude of those synaptic potentials that trigger an up state was apparently larger, given that the sensory evoked potential added up to network recruitment. This is illustrated in Fig. 6E, where averaged responses in Down, In and triggering an up state have been expanded. That panel shows that the peak of the PSP evoked during down and up states coincides (see also Fig. S2B), but not the one that triggers a new up state which occurs later on time, probably as a result of engaging the local circuit. The rise time of spontaneous up states in barrel cortex can be quite steep (Fig. S2D; average of 48 up states), a fact that is obvious as well in the individual up states (Fig. S2E). Therefore, those PSPs that induce the initiation of an up state can result apparently much larger (Fig. S2A, B). In the 13 neurons we recorded from, 53% of the evoked PSPs occurred during up states, 25% were isolated during down states and 22% occurred during down states and triggered up states.

In visual cortex, separation between the two types of PSPs during down states was not necessary, since it was not so common for stimuli to induce an up state. In the case that induced PSPs were followed by an up state, its initiation was generally not as steep as in barrel cortex and therefore the PSPs' peak occurred earlier than the local circuit recruitment.

In the barrel cortex, the average amplitudes of the synaptic potentials measured at the peak of the synaptic potential in the down and up state (Fig. 6E) were: 4.6±2.8 mV (Down), 7.2±3.9 mV (Trigger) and 6.7±2.6 mV (Up) for n = 13 neurons. These amplitudes have been normalized in Fig. 6F (top panel) and the normalized values for each of the 13 neurons are illustrated in Fig. 6F (bottom panel). There it can be seen that out of the 13 neurons, only 3 had an averaged PSP amplitude smaller during the up than the down state. The synaptic potentials evoked during the up states were often followed by the end of the up state, or at least by a partial repolarization of the up state.

### Possible mechanisms of synaptic enhancement during up states

**Figure 7:**
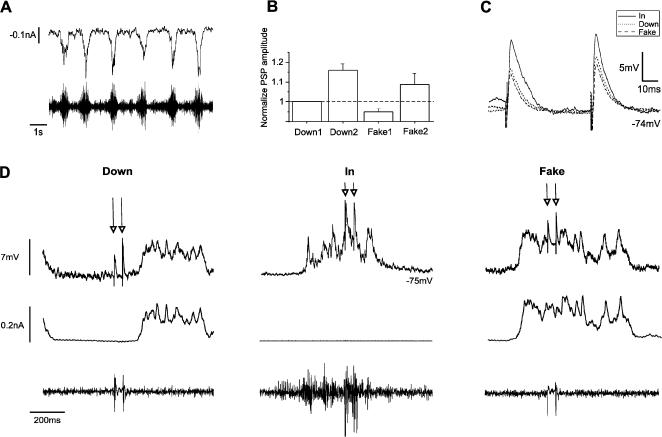
Paired pulse facilitation during up *versus* down states of *fake* oscillations. A. Intracellular recording in voltage clamp during a recording of slow oscillations from a neuron in a slice of the ferret visual cortex (top trace) and multiunit activity in the vicinity of the recorded neuron, illustrating the occurrence of rhytmic activity in the network (bottom trace). B. Relative amplitudes of the first and second PSPs induced during down states *versus* fake up states. Amplitudes are normalized with respect to the amplitude of the first PSP in the down state. Averages correspond to n = 7 neurons. C. Averaged amplitudes of the first and second PSPs in the pair occurring from a neuron (in D) while comparing three situations: down states, synaptically generated up states and *fake* up states. Note that the ones occurring during the actual up states are of larger amplitude. D. Raw traces showing examples from the same neuron as in C with pairs of PSPs ocurring during down states (left), up state (center) and *fake* up state (right). The middle trace is Im (nA) and the bottom trace is the multiunit recording illustrating network activity in the vicinity of the neuron. Note that only in the middle panel, which is a spontaneously generated up state, there is network activity during the up state. The stimuli artifact from the electrical stimulation to activate the PSPs can be seen in the three cases.

To explore which mechanisms could contribute to the increase in amplitude of PSPs during up states we first considered the effect of membrane voltage depolarization typical of up states. By means of single electrode voltage clamp, the current flowing through the membrane during cortical slow oscillatory activity was recorded *in vitro* (Fig 7A). The current trace obtained in that way was subsequently inverted and injected intracellularly into neurons, therefore simulating oscillations by current injection, that we refer to as *fake* oscillations (Fig. 7D, right). In these neurons, paired synaptic potentials were injected during down and during up states, as shown above for spontaneous oscillations (n = 7; Fig 7B). If we consider the amplitude of the first PSP, during the actual up states there was a significant increase in amplitude of intracortical synaptic potentials with respect to the amplitude during down states. On the other hand, during the *fake* up states there was instead a decrease in the first PSPs amplitude (Fig. 7B, C), consistent with a more depolarized state and therefore less driving force for ions passing through glutamate receptors (reversal potential of 0 mV). Both during down states and during *fake* up states there was synaptic facilitation (Fig. 7B). Fig. 7D illustrates a neuron in which pairs of PSPs were evoked during both actual and *fake* oscillations. Note that the first PSP in the pair is clearly increased in amplitude during the real but not during the *fake* oscillation, suggesting that depolarization of the postsynaptic neuron is not involved in the genesis of the phenomenon of potentiation during up states. Furthermore, during the down state and the *fake* oscillation there is paired pulse facilitation, while the two PSPs occurring during the actual up state have the same amplitude.

We injected current into two neurons from the visual cortex in an anesthetized cat *in vivo* in order to induce *fake* oscillations. An intracortical and a thalamocortical synaptic potential were evoked during down states and during up fake states. As it has been described for the *in vitro* situation, no enhancement of the first PSP was observed when synaptic potentials occured during *fake* up states (data not shown).

**Figure 8:**
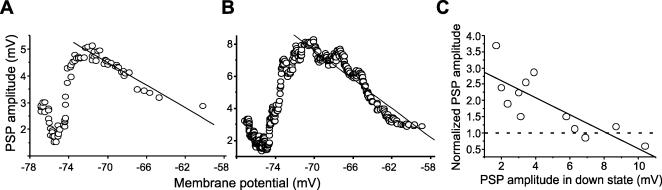
Voltage and amplitude dependence of sensory-evoked synaptic potentials. A. Variation of visually-activated PSPs' amplitude at different membrane potentials corresponding to up and down states in a cat visual cortex neurons. Each point represents the average of 20 sensory responses. A linear fit to those values between −72 and −58 mV (R^2^ 0.95;p<0.0001) illustrates the voltage dependence of the sensory response. B. Variation of whisker-activated PSPs' amplitude at different membrane potentials corresponding to up and down states in a barrel cortex neurons. Each point represents the average of 20 sensory responses. Linear fit (R^2^ 0.92;p<0.0001) as in A. C. Dependence of the normalized air puff induced PSP amplitude in the up with respect to the down state represented against the absolute amplitude of the sensory-evoked potential during the down states. Note that larger PSPs show less increase during up states (R^2^ 0.8, t = 4.216 on 10 degrees of freedom, 2-tailed significance level is 0.0017). One outlier was removed.

Other studies where synaptic potentials during up *versus* down states have been compared report that PSPs decrease in amplitude during up states due to membrane depolarization and the PSPs voltage dependence [12], [23]. We explored the voltage dependence of PSPs evoked by sensory stimulation in visual and barrel cortex. As shown in Fig. 8A and B PSPs in both cortices showed a voltage dependence represented by a linear fit, similar to that reported by others [23]. However, if we look into more hyperpolarized potentials the PSPśamplitude departs from the linearity of the voltage dependence and decreases, revealing an accumulation of values at low amplitudes corresponding to down states. Around −75 mV and towards more hyperpolarized values the amplitude starts increasing again (Fig. 8A, B), probably reflecting a reversal of inhibitory components due to the chloride E_rev_. This finding suggests, as does our data shown above, that not only voltage dependence but other mechanisms participate in establishing the amplitude of PSPs during up and down states.

**Figure 9:**
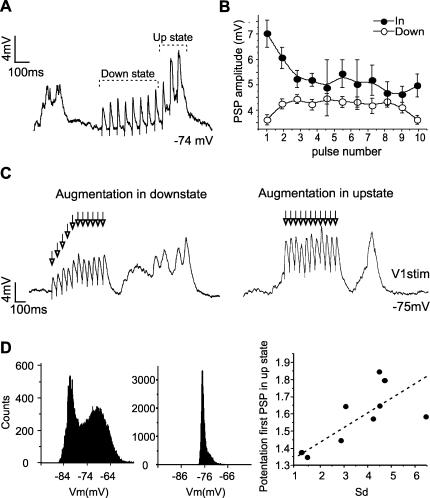
Synaptic enhancement with trains of electrical stimuli. A. Intracortical synaptic potentials activated by a 20 Hz train of electrical stimulation *in vivo*. The first 8 PSPs occurred during the down state and the following ones coincided with an up state. Note that the ones during the down state display synaptic facilitation, but their amplitude increased even further when occurring during the up state. B. Averaged PSPs amplitudes occurring during up (*In*) and down (*Down*) states for the same neuron as in A. Each point represents an average of 8–16 PSPs. Error bars are s.e.m. C. Repetitive electrical activation of intracortical synaptic potentials *in vivo* during down states (left) and during an up state (right). D. Distributions of the membrane potential values measured in a time window of 60 s and with a sampling rate of 10 KHz in a neuron during high spontaneous activity (left panel; s.d. 6.28) and another neuron with less spontaneous activity (middle panel; s.d. 1.62). Correlation between the standard deviation of the membrane distribution and the amplitude increase of the first PSP in the pair (n = 9 neurons; right panel).

Another factor that should be taken into account when comparing sensory-evoked synaptic potentials in up *versus* down states is the amplitude of the evoked response. Our results revealed for PSPs evoked by whisker stimulation a significant relationship between the amplitude of the PSPs during the down states and their degree of its enhancement during up states, such that synaptic potentials of lesser amplitude would be enhanced to a greater extent (Fig. 8C). This could be an important element to explain the discrepancy between the findings reported in different studies [23], [25] and this one. Next, we considered whether reverberatory activity during up states could activate synapses repeatedly thus inducing an increase in PSP's amplitude that would underlie the one observed during up states. In a total of 10 neurons (n = 7 *in vitro* and n = 3 cat *in vivo*), trains of 10–12 electric shocks at different frequencies were given. In neurons recorded *in vitro*, trains of shocks at 20 Hz given during down states induced synaptic augmentation to 1.65 times the size of the first PSP, increase that remained in a plateau for 6–7 pulses before starting to decay (Fig. 9B, empty circles). Therefore, in order to obtain an increase in amplitude of the order that we have observed during up states *in vitro* for intracortical connections, the presynaptic terminals could have been activated 3–4 times at 20 Hz. When the same synaptic potential was activated during the up state, it increased to the same value or even higher from the first shock (Fig. 9A, B), and no further enhancement was observed with the train of repetitive presynaptic stimulation (Fig. 9C), or eventually some depression (Fig. 9B, filled circles). This suggests that whatever mechanisms are activated by the up states in the synapse, they are saturating the mechanisms of synaptic short term synaptic enhancement.

For the recordings in the visual cortex of the cat *in vivo*, we quantified ongoing activity by determining the standard deviation of the membrane potential [9], [15]. We found a significant correlation (R^2^ = 0.69; p = 0.04) between the standard deviation of the membrane potential values (Fig. 9D, left and center panels) and the mean increase of the first PSP during up states (n = 9) (Fig. 9D, right panel), indicating larger synaptic enhancement during up states in more active cortical networks.

## Discussion

Our results demonstrate that intracortical synapses in an active, oscillatory cortical network *in vitro* have a tendency to augment rather than to depress if repeatedly activated 10–15 times. Longer trains would drive the synapses to depress, although less in active than in silent cortical slices [15]. The same synaptic connections in silent slices (without spontaneous activity) increase less and display depression earlier in the train. Early synaptic depression and absence of synaptic facilitation was more extreme in silent slices maintained in a 2mM [Ca]_o_ ACSF (‘classical ACSF’; see Methods) than in those in 1–1.2 mM [Ca]_o_ ACSF (‘in vivo-like ACSF’; Fig. 1). This observation agrees with the well known fact that conditions in which there is an increase in probability of neurotransmitter release i.e. higher [Ca]_o_, entail a larger synaptic depression [15], [31], probably as a result of the faster depletion of a readily releasable vesicle pool that occurs with repetitive presynaptic activation [32]–[34]. On the other hand, lower Ca^2+^ in the solution and the subsequent decrease in the probability of release typically reduces synaptic depression [35], [36]. Apart from the effect due to differences in ionic concentrations, slices maintained in the same ACSF (*in vivo-like*) consistently displayed more synaptic enhancement and less depression when there was ongoing activity in the slice than when the network was silent (Fig. 1).

Protocols of paired pulses with different time intervals in between revealed a temporal course of paired pulse synaptic facilitation that followed the same trend, with higher and longer lasting facilitation in active than in silent slices (Fig. 2). These findings are in agreement with previous data reporting that high levels of neuronal activity in the thalamocortical network promote synaptic augmentation and decrease depression [11], [12], [14], [15], [37]. What mechanisms may be responsible for this influence of activity on synaptic plasticity? A different steady state of the neurotransmitter release system in an active network [11], [14], [15] or a decrease in extracellular calcium levels during activity [13], [38] may affect the release of neurotransmitter and as a result, synaptic short term plasticity [36]. The extent to which the rhythmic activation of the network exerts a different influence on synaptic plasticity than a stochastic activation remains to be studied.

Once we had established that activity in the network modifies short term plasticity, we explored how the occurrence of up *versus* down states affected synaptic transmission and plasticity induced by paired pulses.

### Enhancement of electrically and visually evoked synaptic responses during up states

One of the main results from this study is that electrically evoked synaptic potentials both intracortical (*in vitro* and *in vivo*) and thalamocortical (*in vivo*) showed a significant increase in amplitude during up states with respect to down states in the visual cortex. The same was true for visually and whisker evoked synaptic potentials. An identical stimulus (visual or whisker) evoked a larger synaptic potential during the up states than during down states. The observation does not seem surprising since during up states the thalamocortical loop is in a more depolarized state and therefore the same visual or tactile stimulus should evoke a larger response. Indeed, suprathreshold visually evoked responses have been found to be significantly increased in up with respect to those during down states as reported in a recent paper [39], and this is in agreement with the finding of a positive correlation between the amplitude of visual responses and the preceding value of spontaneously changing membrane potential in visual cortex [24].

However, these results are opposite to the ones reported for sensory evoked responses in the rat barrel cortex [23], [25], where sensory or electrically induced PSPs have been reported to be diminished during up states, and in cortical areas 5 and 7 following single axon stimulation [13]. In our results we point out a possible explanation of the discrepancy observed with whisker evoked responses occurring in up and down states. We observed that out of those sensory evoked potentials occurring during down states in the barrel cortex, approximately 50% do trigger the beginning of a new up state. If those PSPs would be averaged with the rest of the ones occurring during down states and the amplitude measured at the peak, the resulting amplitude would then include network recruitment and therefore it would surpass the purely sensory evoked response during the down state. Thus, when compared with sensory evoked responses during up states, the ones occurring during down states could appear to be larger. Indeed, in our recordings from rat barrel cortex, if we average together all PSPs amplitudes evoked in down states (“Down” plus “Trigger”) and compare with those evoked during up states (“Up”), no statistical differences between PSPs them were observed (down: 5.9±3 mV and up: 6.7±3 mV, n = 13).

The decrease of PSṔs amplitude during up states has been attributed to the voltage dependence of glutamatergic excitatory responses [13], [23]. By intracellularly injecting current that simulates up states we demonstrate that indeed depolarization results in a decrement of the evoked synaptic potentials, which is opposite to what happens during an actual up state (Fig. 7). The voltage dependence of visual and whisker-induced sensory responses that we found (Fig. 8A) is similar to the one reported by others (Fig. 3 in Petersen et al., 2003), however the evolution of the synaptic potential in a larger voltage range reveals a departure from voltage dependence and low PSPs' amplitudes for hyperpolarized potentials.

The disparity between studies could be due to different reasons: 1) different amplitude of sensory evoked potentials (see Fig. 8C); 2) stimulating one whisker versus several whiskers, 3) averaging PSPs that trigger an up state together with those occurring during down states and comparing them with PSPs in up states, 4) different cortical areas, species, or specific synaptic connections that behave differently [40], [41]. Presynaptic recruitment and conductance changes during up states are other possible causes of variation in PSP's amplitude during up states that will be discussed below.

### Possible mechanisms mediating synaptic enhancement during up states

In our study we have first considered an obvious difference between up and down states, which is the membrane potential depolarization that occurs during up states. The recorded current in voltage clamp underlying membrane oscillations was reinjected into neurons in order to simulate realistic oscillations with synaptic noise. ‘Fake’ oscillations induced by current injection had indeed an effect on the PSPs amplitude. However this effect was the one expected from a membrane depolarization, which is a scaling effect towards a smaller size due to the decreased driving force for glutamatergic excitation, and it implied no changes in the relative amplitude of the second with respect to the first PSP in the pair. Therefore, we can conclude that the depolarization *per se* does not cause or participate in the synaptic increase observed during up states, since the change that would cause would be in the opposite direction. The effect of amplitude decrease of the synaptic potential secondary to depolarization is necessarily added to the synaptic changes, and therefore our measurements of enhancement are slightly understimated since the correction of synaptic potential amplitude due to changes in membrane potential was not taken into account (see Results).

Changes in conductance during up and down states both *in vivo* and *in vitro* have been greatly discussed in recent experimental and theoretical studies. An increased conductance of different degrees has been generally associated with up states [21], [42]–[45], resulting in a potential divisive gain control of neuronal responses [45], [46]. Indeed, decreased synaptic responses during up states has been mainly attributed to an increased conductance during high synaptic bombardment [13], [23]. Contrary to these, a similar conductance during up states and down states [47] or even a decreased overall conductance occurring during up states has also been reported, as a result of sparse synaptic activity plus anomalous rectification [48]. A decrease in conductance would imply an increase in the amplitude of the incoming synaptic potentials during up states. Indeed, an increase in R_IN_ during up states of 10–15% [48] would increase the amplitude of the PSPs in the same proportion, which is less than the increment of the first PSP amplitude that we detected for intracortical connections (around 160% increment; Figs. 3 and 4). Besides, if a decrease in conductance would be the only intervening factor, its effect should be equal for both first and second PSPs in the pair, and therefore both PSPs would display the same paired pulse facilitation as during down states but multiplied by a certain factor. Our results though describe a different scenario: a significant increase in the amplitude of the first PSP of the pair during up states and a relation with the second PSP that differs for intracortical and thalamocortical synapses. However, the changes on the first and second PSP in the up with respect to the down state is never one of a multiplicative effect. Therefore, even when a decrease in conductance would explain in part the observed synaptic enhancement during up states, other mechanisms seem to be at play that could explain the differential effects observed in the first and second PSPs in up *versus* down states. Differences in PSP's amplitude reported during the 200 ms of down states that followed the end of up states *in vivo* (so called ‘End’ period, Fig.3) also disagree with a change in conductance-based mechanism. Given that during the ‘End’ period the conductance is that of a down state, the remaining synaptic enhancement (Fig. 4) must involve an additional mechanism inducing synaptic plasticity.

Another possibility that explains the greatly larger amplitude of synaptic potentials when overriding the up states is that the electric shock that causes presynaptic activation could recruit a larger number of presynaptic terminals during the up states, given that the activation is affecting the whole thalamocortical loop [49]–[51]. During up states neurons are more depolarized and therefore closer to firing threshold [39]; as a result, more neurons could be recruited with the same stimulus given during an up than during a down state. Even when electric shocks seem to activate axons rather than somas, axonal threshold could be also be lowered by increased potassium levels during up states [52]. Thus, when electrical or natural stimuli are given (visual, whisker) the same stimulus probably recruits a larger number of presynaptic neurons along the ascending pathway, therefore resulting in larger PSPs during up than during down states. If that is the case, and if we consider that up or activated states are somehow equivalent to alert states (see below) it would imply that during awake states the transmission of a natural stimulus is more efficient and secure, as we observed during up states.

Some of our observations though seem to argue though against this possibility, or at least against it as a unique explanation. We observed that intracortical PSPs evoked during the 200 ms following the occurrence of the up states (‘End’ period, see Results) were still greatly increased in amplitude *in vivo* to about 1.2 times the amplitude of the PSPs occurring during downstates (Fig. 4). Therefore, the effect that the up state has on the synapses has a time course of decay, and it does not vanish with the sudden start of the down state as it would if due to differences in presynaptic recruitment. Also in thalamocortical PSPs the facilitating effect of the up state still remains 200 ms into the down state, with an average amplitude increase of twice the PSP amplitude in the down state. In this period that follows an up state thalamocortical connections display a consistent paired pulse depression, which adds evidence towards phenomena of synaptic plasticity rather than variability in presynaptic recruitment.

As we found *in vitro* (Fig.1C), repetitive activation (10 pulses) of intracortical monosynaptic potentials in the vicinity of the recorded neuron *in vivo* usually displayed augmentation (Fig. 1C, 9A–C). The maximum PSP's amplitude was reached with 3–4 pulses at 20Hz. This maximum amplitude was similar to the one reached if the PSPs were activated during the up states. This finding suggests that the up states have an effect similar to repetitive stimulation on synaptic transmission. This effect could be mediated through synaptic activation at high frequencies (15–80 Hz) during the up states [53], [54]. During up states [Ca]_o_ decreases [38], and synaptic enhancement could occur by spontaneous activation of the presynaptic neurons during the up states, causing in them a depolarization that would generate larger PSPs [55] and a calcium increase in the synaptic terminals [56].

That up states may be comparable to the alert cortical functional state is suggested by intracellular recordings during alert states [57] and during awake-sleep transitions that reveal a similar Vm during up and awake states, and the progressive appearance of down states with drowsiness, those becoming more frequent during periods of slow wave sleep [3], [51]. Meanwhile, membrane voltage at a depolarized value and the high noise resulting from impinging synaptic inputs remains alike during the alert state and during up states of slow wave sleep. Furthermore, oscillations at high frequencies (beta and gamma) that have been repeatedly associated with sensory and cognitive functions -and therefore to alert states- also occur during up states in slow wave sleep [53], [54]. This may suggest that synaptic transmission and plasticity in the visual cortex during up states, more secure and efficient due to a larger amplitude and lesser short term plasticity, is similar to transmission during the awake state.

## Supporting Information

Figure S1 Paired- pulse mono and polysynaptic thalamocortical synaptic potentials in cat visual cortex during up and down states. A. Normalized amplitudes for monosynaptic PSPs separated by a 50 ms interval during down states, up states, and during 200 ms following an up state (end). This representation includes n = 9 neurons that had a latency under 3 ms. B. Example of a monosynaptic thalamocortical PSP. C. Normalized amplitudes for polysynaptic PSPs separated by a 50 ms interval during down states, up states, and during 200 ms following an up state (end). This representation includes n = 6 neurons that had a latency between 3 and 3.6 ms. D. Example of a polysynaptic thalamocortical PSP.
(6.44 MB TIF)

Figure S2 Slow rhythmic activity and whisker evoked responses in rat barrel cortex during down and up states. An illustrative example. A. Raw traces of whisker evoked responses in three different periods: left, sensory evoked responses occuring during down states and not triggering the initiation of a new up state. Middle, sensory evoked responses occuring during down states and triggering the initiation of a new up state. Right, sensory evoked potentials occuring during up states. B. Overlapped averages of 20 sensory evoked responses in each case: whisker evoked responses occuring in the down states, in the down state and triggering an up state, and during an up state (as in A from left to right respectively). The discontinous lines mark the initiation of the sensory evoked response (left) and its peak in down and up state. Notice the large difference in amplitude between those occurring during down states that do not trigger a new up state and those that do. C. Top trace, unfiltered field potential reflecting population activity. Bottom trace, five spontaneous up states. No sensory stimulation has been delivered. D. Average of the first 230 ms of 48 subthreshold up states as the ones in E. This is an example illustrating a steep rise of the membrane potential during the initiation of the up states. E. Top trace, unfiltered field potential recording reflecting population activity. Bottom trace, five spontaneous up states, the neuron has been kept subthreshold. No sensory stimulation has been delivered.
(0.14 MB PDF)
