# Supplementary figures and images for: Synaptic Transmission and Plasticity in an Active Cortical Network
Source: PLoS One. 2007 Aug 1;2(8):e670. doi: 10.1371/journal.pone.0000670 (PMC1925142; doi:10.1371/journal.pone.0000670)

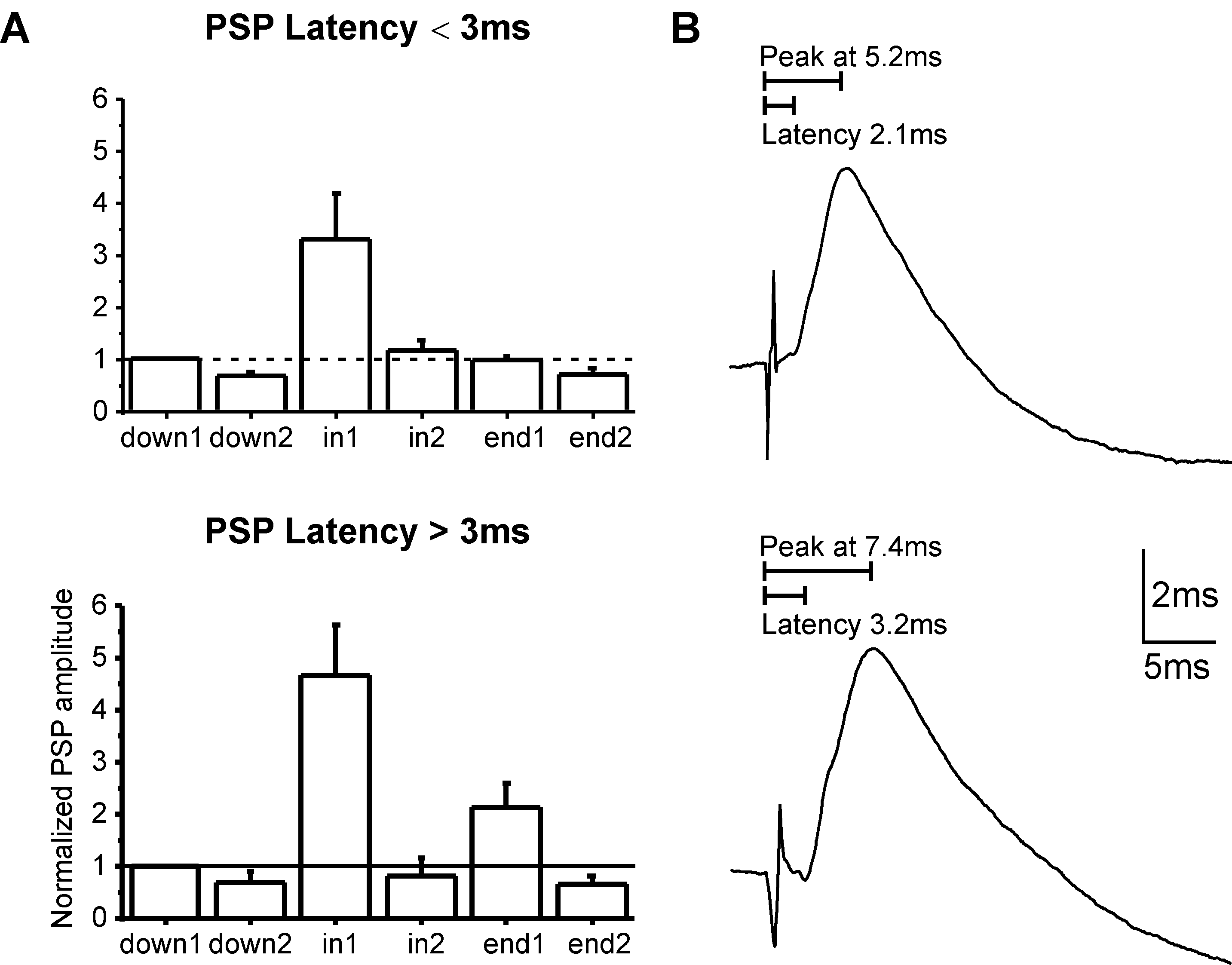

Supplement: Figure S1 — Paired- pulse mono and polysynaptic thalamocortical synaptic potentials in cat visual cortex during up and down states. A. Normalized amplitudes for monosynaptic PSPs separated by a 50 ms interval during down states, up states, and during 200 ms following an up state (end). This representation includes n = 9 neurons that had a latency under 3 ms. B. Example of a monosynaptic thalamocortical PSP. C. Normalized amplitudes for polysynaptic PSPs separated by a 50 ms interval during down states, up states, and during 200 ms following an up state (end). This representation includes n = 6 neurons that had a latency between 3 and 3.6 ms. D. Example of a polysynaptic thalamocortical PSP. (6.44 MB TIF) [file pone.0000670.s001.tif]

**A**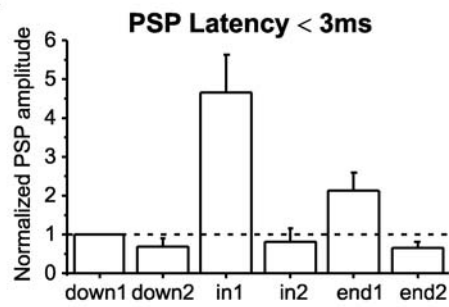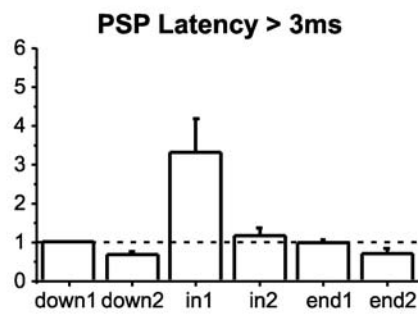**B**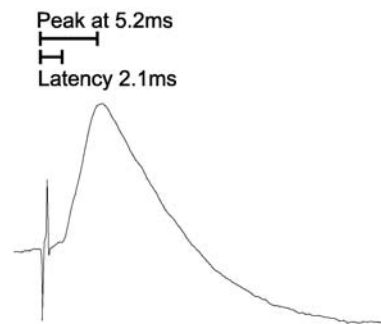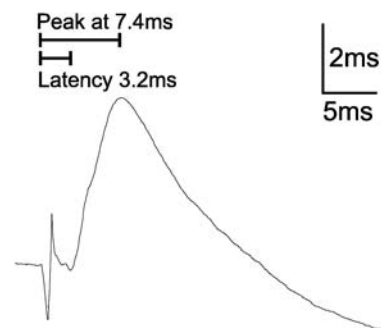

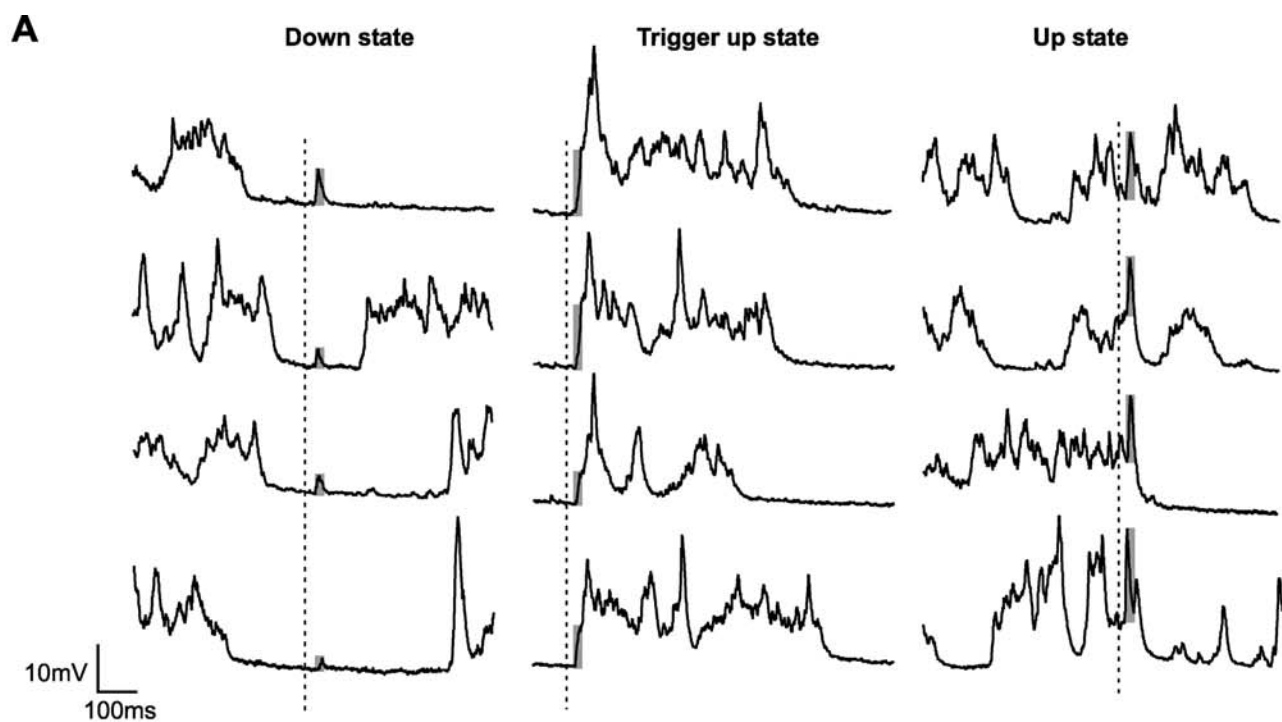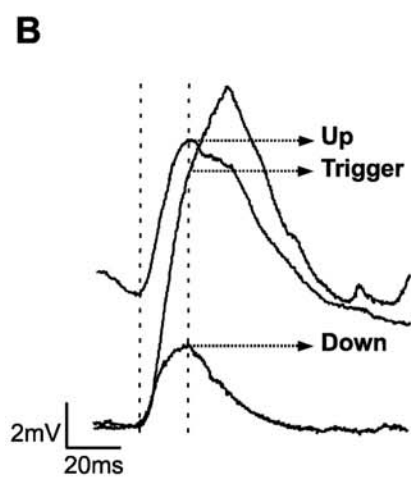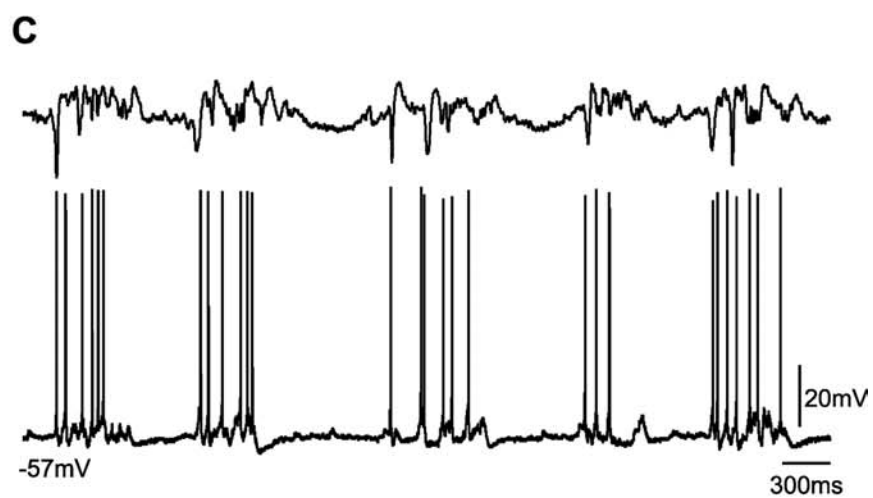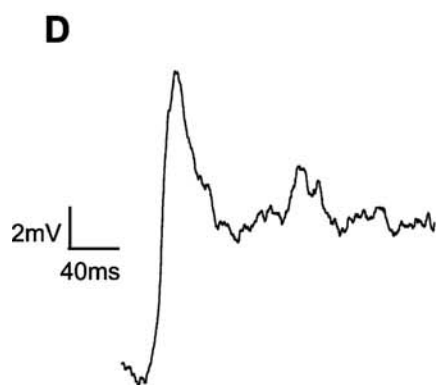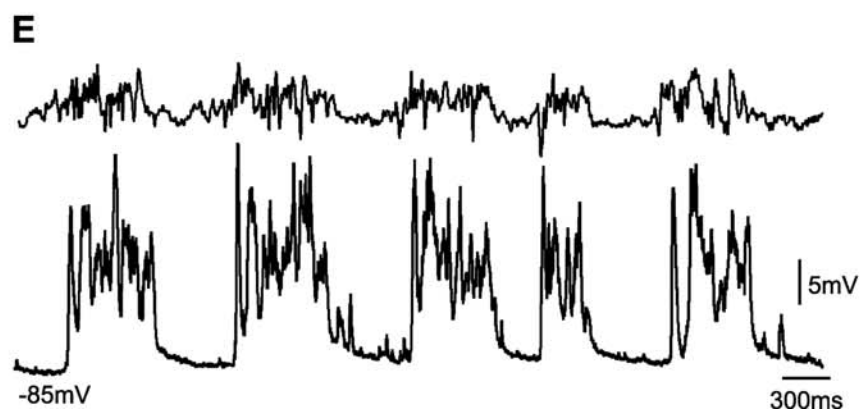

Supplement: Figure S2 — Slow rhythmic activity and whisker evoked responses in rat barrel cortex during down and up states. An illustrative example. A. Raw traces of whisker evoked responses in three different periods: left, sensory evoked responses occuring during down states and not triggering the initiation of a new up state. Middle, sensory evoked responses occuring during down states and triggering the initiation of a new up state. Right, sensory evoked potentials occuring during up states. B. Overlapped averages of 20 sensory evoked responses in each case: whisker evoked responses occuring in the down states, in the down state and triggering an up state, and during an up state (as in A from left to right respectively). The discontinous lines mark the initiation of the sensory evoked response (left) and its peak in down and up state. Notice the large difference in amplitude between those occurring during down states that do not trigger a new up state and those that do. C. Top trace, unfiltered field potential reflecting population activity. Bottom trace, five spontaneous up states. No sensory stimulation has been delivered. D. Average of the first 230 ms of 48 subthreshold up states as the ones in E. This is an example illustrating a steep rise of the membrane potential during the initiation of the up states. E. Top trace, unfiltered field potential recording reflecting population activity. Bottom trace, five spontaneous up states, the neuron has been kept subthreshold. No sensory stimulation has been delivered. (0.14 MB PDF) [file pone.0000670.s002.pdf]
